# A case of Barrett’s esophageal adenocarcinoma and severe scoliosis with successful salvage esophagectomy after definitive chemoradiotherapy

**DOI:** 10.1186/s40792-023-01776-8

**Published:** 2023-11-24

**Authors:** Daisuke Taguchi, Kotaro Yamashita, Kota Momose, Koji Tanaka, Tomoki Makino, Takuro Saito, Kazuyoshi Yamamoto, Tsuyoshi Takahashi, Yukinori Kurokawa, Kiyokazu Nakajima, Hidetoshi Eguchi, Yuichiro Doki

**Affiliations:** Department of Gastroenterological Surgery, Osaka University Graduate School of Medicine, 2-2-E2 Yamadaoka, Suita City, Osaka 565-0871 Japan

**Keywords:** Esophageal cancer, Barrett’s esophagus, Scoliosis, Salvage surgery

## Abstract

**Background:**

Severe scoliosis can cause reflux esophagitis, and is a risk factor for Barrett's adenocarcinoma of the esophagus. Severe scoliosis is associated with respiratory dysfunction, impaired operative tolerance, and anatomical difficulty in surgical manipulation, and is, therefore, considered a high surgical risk. In this report, we describe the case of a young patient with Barrett's esophageal adenocarcinoma with severe scoliosis who underwent salvage surgery after radical chemoradiotherapy.

**Case presentation:**

The patient was a 39-year-old male. Although he had severe scoliosis and paraplegia of both lower limbs since childhood, he was independent in activities of daily living. His previous doctor, who diagnosed the esophageal cancer, determined that surgery was not indicated due to the coexistence of severe scoliosis, so he underwent chemoradiotherapy with curative intent. After chemoradiotherapy, the patient was referred to our hospital for a second opinion because of a tumor remnant. After various additional examinations, thoracoscopic and laparoscopic subtotal esophagectomy for esophageal cancer was performed, along with ante-thoracic route reconstruction using a narrow gastric tube. Although the patient had symptoms associated with postoperative reflux, he recovered well overall and was discharged home about 4 weeks after surgery.

**Conclusions:**

We report the case of an esophageal cancer patient with severe scoliosis at high surgical risk who underwent successful minimally invasive esophagectomy.

## Background

Severe scoliosis is known to cause reflux esophagitis [1], and is associated with respiratory dysfunction [2] and impaired operative tolerance. In addition, esophageal cancer patients with severe scoliosis are considered to experience more frequent surgical difficulties when undergoing esophagectomy because of anatomical variations. There are few reports of surgical cases of esophageal cancer with coexisting scoliosis. In addition, salvage esophagectomy after definitive chemoradiotherapy is known to be one of the most difficult surgical procedures [3], with a high incidence of postoperative complications [4]. Here, we report a case of Barrett’s esophageal adenocarcinoma in a young patient with severe scoliosis who underwent definitive chemoradiotherapy followed by salvage esophagectomy.

## Case presentation

**Fig. 1:**
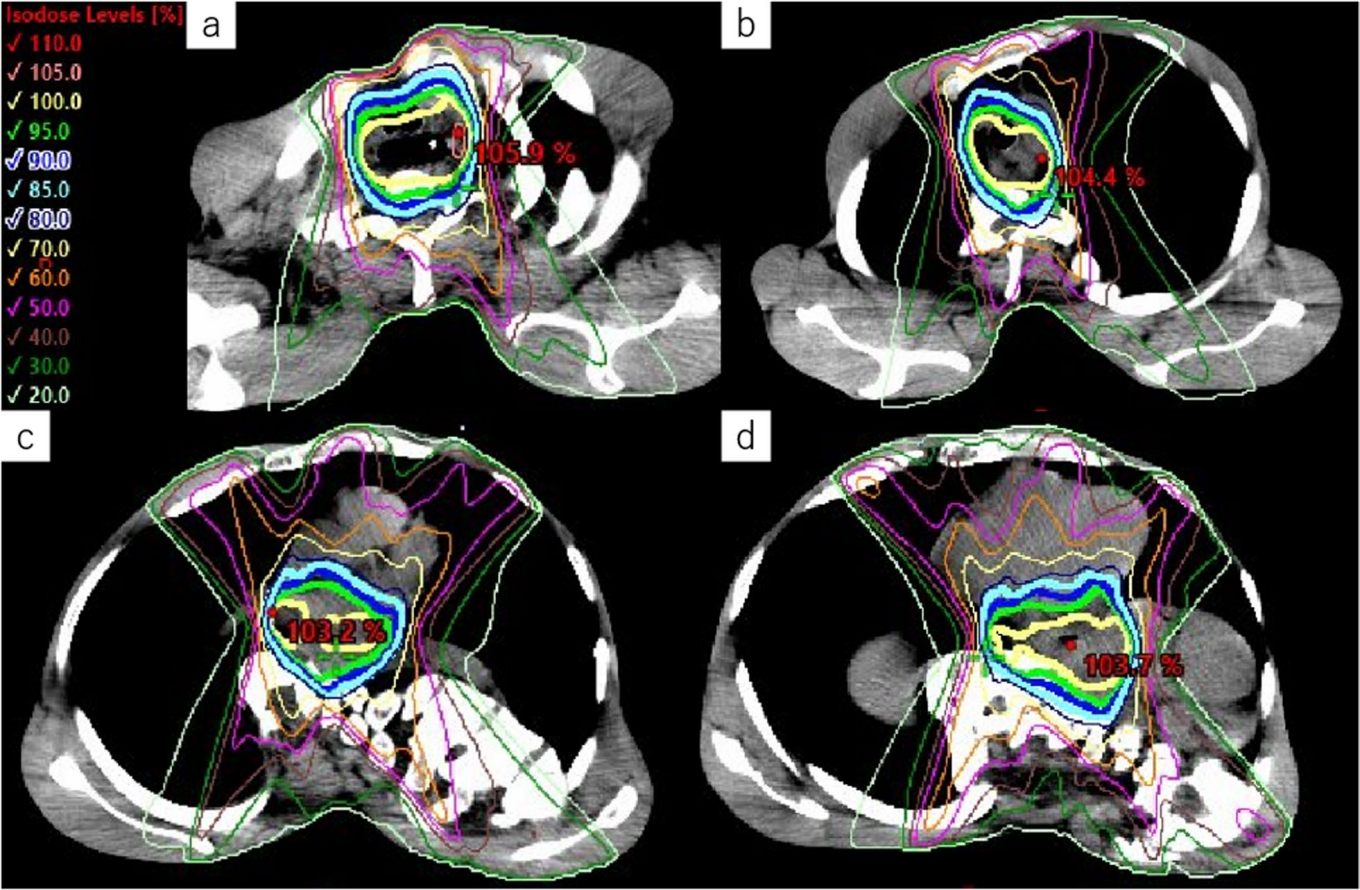
Radiation dose distribution maps: radiation was administered to the upper (**a**, **b**), middle (**c**), and lower thoracic esophagus (**d**), where the tumor was located before the intervention. A total of 61.2 Gy of radiation was administered, with up to 41.4 Gy/23 Fr irradiated to the area, including prophylactic irradiation, and the subsequent 19.8 Gy/11 Fr targeted to the tumor

The patient was a 39-year-old male. He noticed heartburn and dysphagia 11 months before eventual referral to our hospital. The symptoms persisted, and he, therefore, consulted his local doctor 6 months later, and underwent an upper gastrointestinal endoscopy, which revealed Barrett's esophagus and adenocarcinoma of the esophagus. After starting proton pump inhibitor (PPI) treatment, he was referred to his previous hospital for esophageal cancer treatment. Since he was judged to be ineligible for surgery due to severe scoliosis and restricted ventilation, he received curative-intent chemoradiotherapy (4 courses of CF [cisplatin and fluorouracil] with radiotherapy [61.2 Gy/34 fractions]) (Fig. 1). After the treatment, he was referred to our hospital for a second opinion regarding additional treatment due to evidence of remnant cancer.

Medical history: paraplegia in both lower limbs, spinal cord tumor (at age 4).

Family history: none noted.

Physique on admission: height 141 cm, weight 38.3 kg, body mass index 19.3.

Respiratory function test: Before chemoradiotherapy, vital capacity (VC) 1.54 l, %VC 48.6%, forced expiratory volume in 1 s (FEV1.0) 1.46 l, FEV1.0% 94.8%; after chemoradiotherapy, VC 1.51 l, %VC 47.2%, FEV1.0 1.32 l, FEV1.0% 87.4%.

**Fig. 2:**
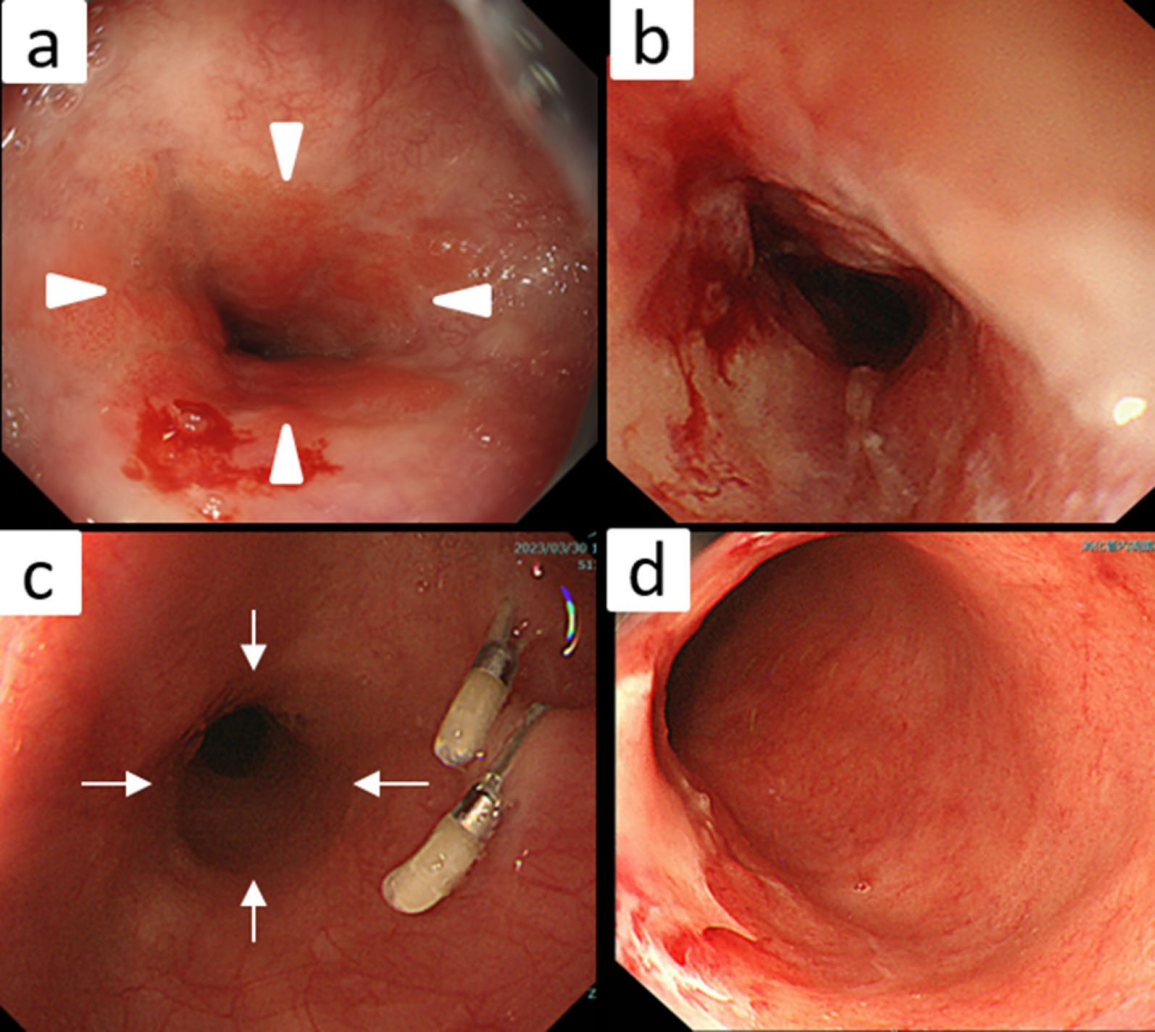
Upper gastrointestinal endoscopy images: **a** Before chemoradiotherapy, the SCJ was observed 20 cm from the incisors (arrowheads). A marking clip was placed at the same site. **b** Circumferential mucosal irregularity and stenosis were observed between 22 and 29 cm from the incisors. **c** After chemoradiotherapy, the SCJ was identified 3 cm distal from the marking clip (arrows). **d** Residual tumor was observed 24 cm from the incisors

Upper gastrointestinal endoscopy: Before chemoradiotherapy, the squamocolumnar junction (SCJ) was observed 20 cm from the incisors. A marking clip was placed at the same site. Circumferential mucosal irregularity and stenosis were observed between 22 and 29 cm from the incisors. The esophagogastric junction was identified 35 cm from the incisors. The lesion was biopsied and diagnosed as adenocarcinoma. After chemoradiotherapy, the SCJ was found 3 cm distal from the marking clip, suggesting that Barrett's esophagus had regressed due to chemoradiotherapy and PPI treatment. The residual tumor was observed 24 cm from the incisors (Fig. 2).

**Fig. 3:**
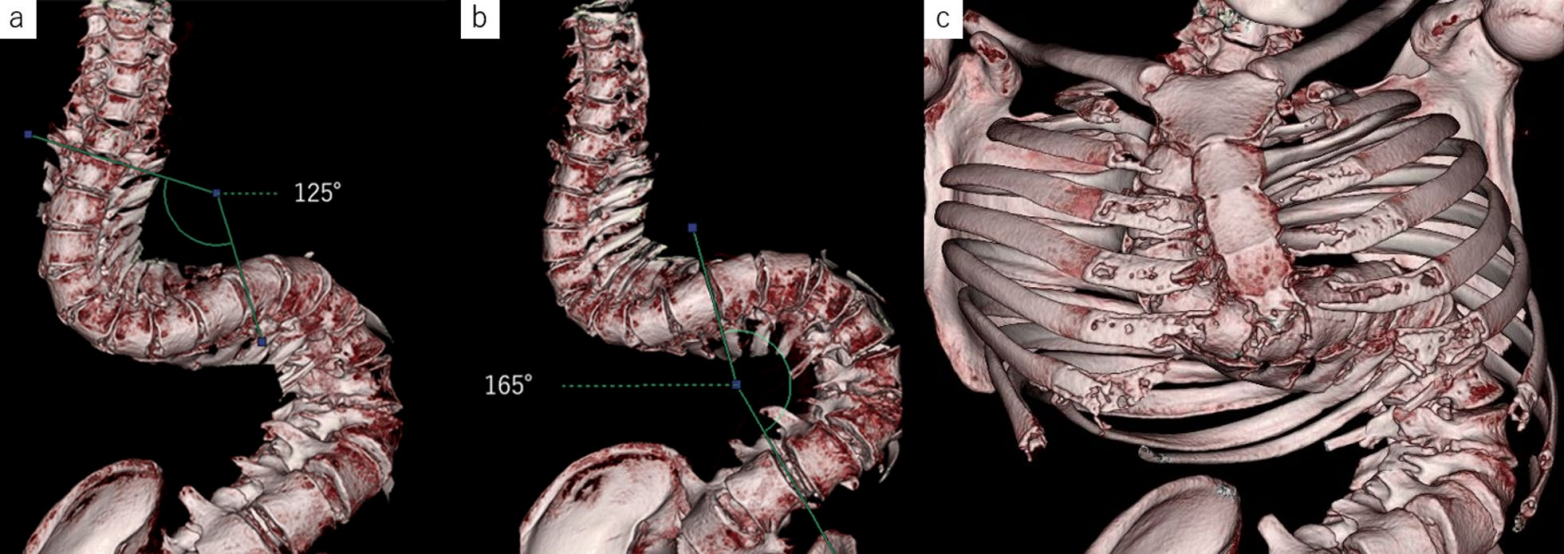
The patient had severe scoliosis. The spinal curve was convex to the right at the level of the middle thoracic esophagus, and convex to the left at the level of the lower esophagus, with Cobb angles of 125° and 165°, respectively (**a**, **b**). 3D-CT image of the spine and ribs shows the narrowing of the rib cage (**c**)

**Fig. 4:**
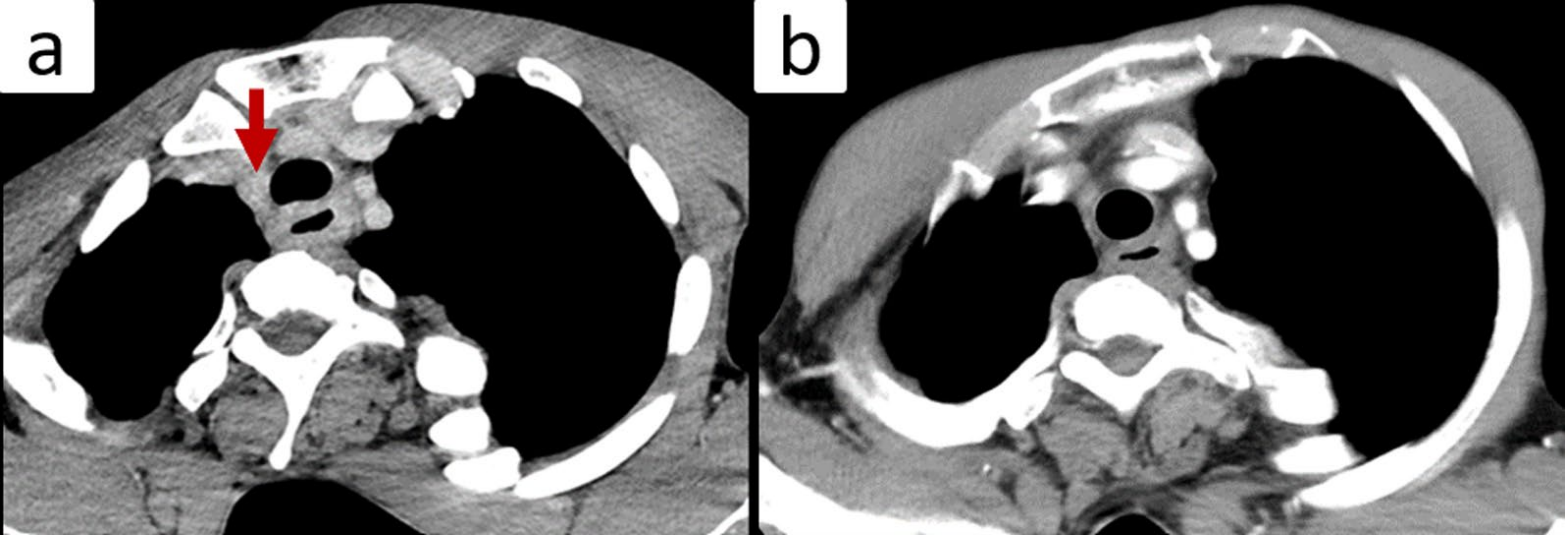
Contrast-enhanced CT images: **a** Before chemoradiotherapy, there was thickening of the esophageal wall from the upper to middle thoracic esophagus, and enlarged lymph nodes were present around the right recurrent nerve (arrow). **b** After chemoradiotherapy, CT showed reduced esophageal wall thickening with no enlarged lymph nodes

Contrast-enhanced CT of the abdomen: The patient had severe scoliosis. The spinal curve was convex to the right at the level of the middle thoracic esophagus, and convex to the left at the level of the lower esophagus, with Cobb angles of 125° and 165°, respectively (Fig. 3). Before chemoradiotherapy, there was thickening of the esophageal wall from the upper to middle thoracic esophagus. There were enlarged lymph nodes around the right recurrent nerve. After chemoradiotherapy, CT showed reduced wall thickening and no enlarged lymph nodes (Fig. 4).

**Fig. 5:**
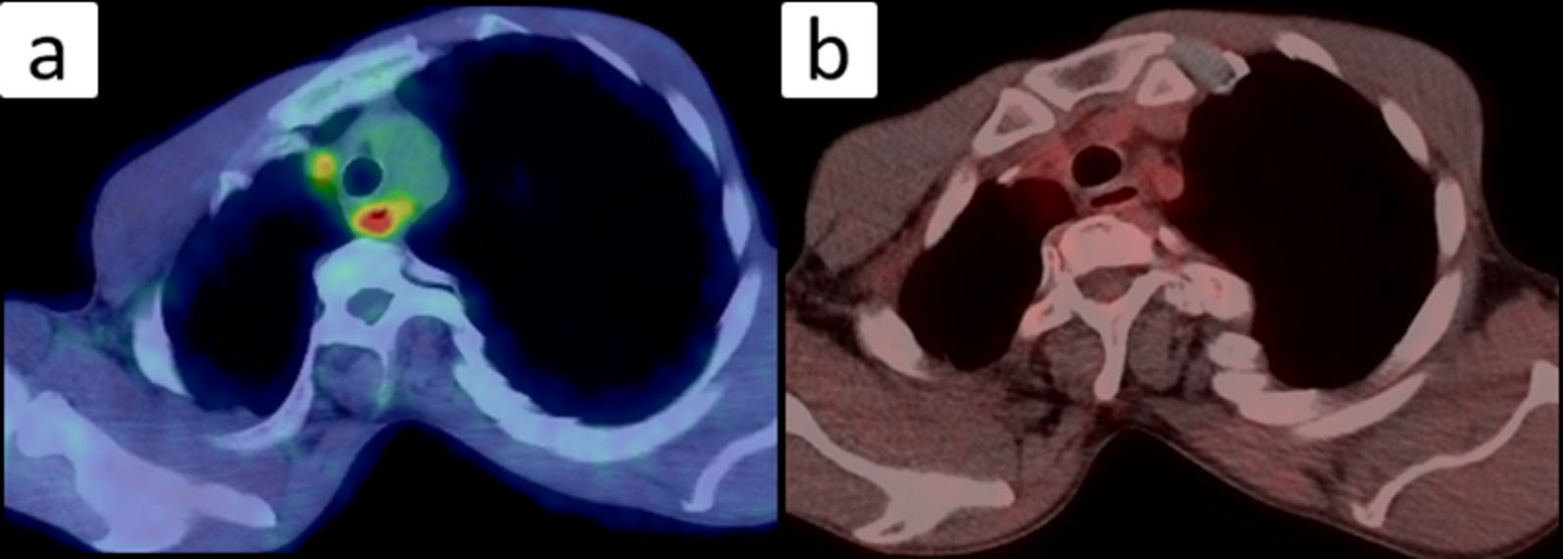
PET–CT images: **a** Before chemoradiotherapy, abnormal 18F-FDG accumulation was seen from the upper to middle thoracic esophagus, with an SUV max of 11.9. Mildly abnormal 18F-FDG accumulation was also seen in the lymph nodes around the right recurrent nerve. **b** After chemoradiotherapy, abnormal 18F-FDG accumulation remained only in the middle thoracic esophagus, with an SUV max of 3.87. Abnormal 18F-FDG accumulation in the lymph nodes had disappeared

Positron emission tomography–CT: Before chemoradiotherapy, abnormal accumulation of 18F-fluorodeoxyglucose (18F-FDG) was seen from the upper to middle thoracic esophagus, with a maximum standardized uptake value (SUV) of 11.9. Mildly abnormal accumulation was also seen in the lymph nodes around the right recurrent nerve. After chemoradiotherapy, abnormal accumulation of 18F-FDG remained only in the middle thoracic esophagus, with an SUV max of 3.87. The abnormal accumulation in the lymph nodes around the right recurrent nerve had disappeared. No other abnormal accumulation was observed (Fig. 5).

**Fig. 6:**
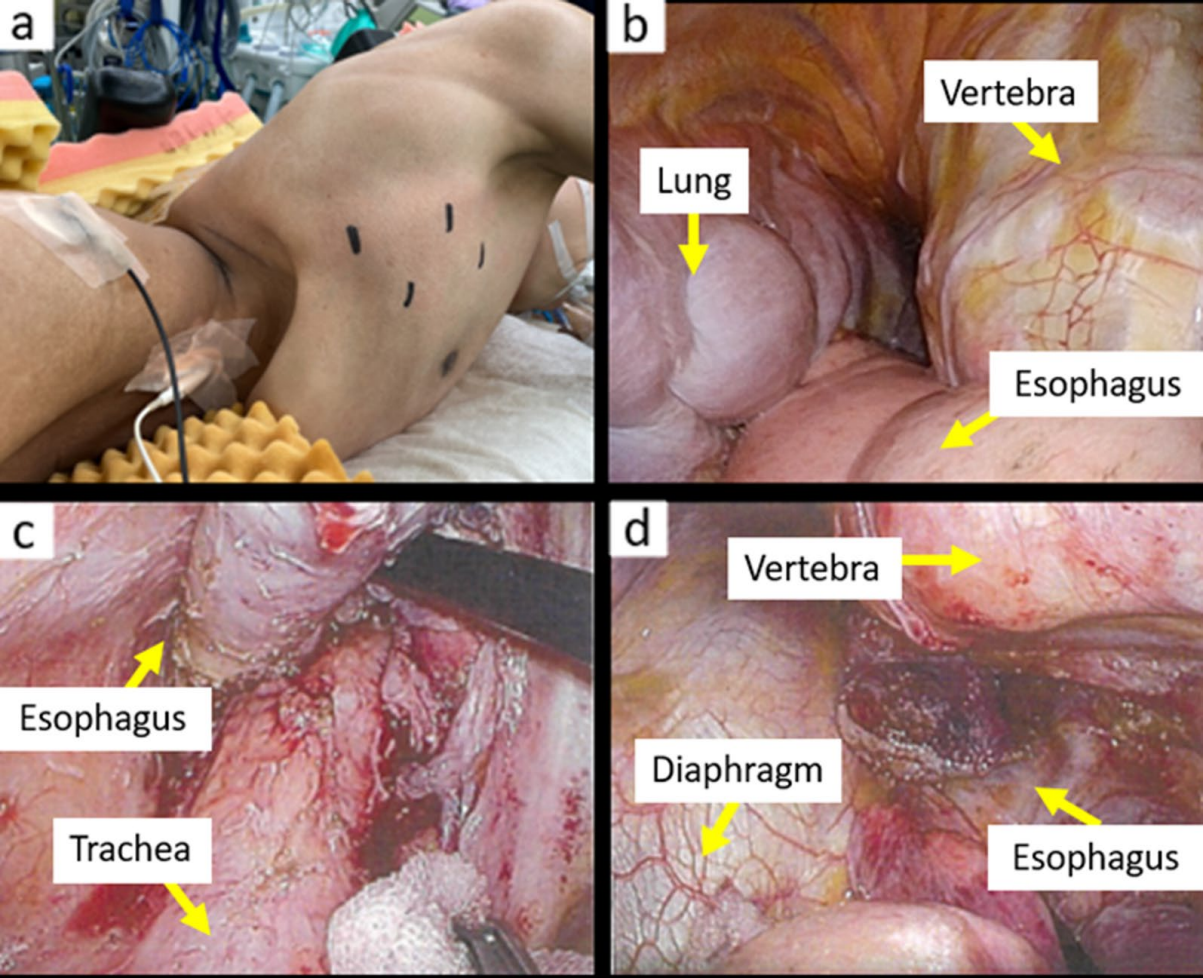
Intraoperative images: **a** Thoracic procedure was performed with the patient in the left lower semi-prone position. We selected the sixth intercostal posterior axillary line as the location for insertion of the first port and subsequently inserted three ports as marked. **b** Thoracoscopic view of the lower mediastinum. **c** There was marked tissue fibrosis in upper mediastinal area, especially on the right side, which was thought to be caused by the effects of chemoradiotherapy on the tumor. **d** Free space was narrow, and forceps operation was limited by the convex curve of the thoracolumbar spine when dissecting the lower thoracic esophagus

Surgical findings: The patient underwent thoracoscopic subtotal esophagectomy involving gastric tube creation by hand-assisted laparoscopy with a flexible endoscope, followed by reconstruction with a narrow gastric tube via the anterior chest wall, and finally gastrostomy. Due to the minimal space in the thoracic cavity, there were few positions for port insertion. We selected the sixth intercostal posterior axillary line as the location for insertion of the first port. We subsequently inserted three ports as marked in Fig. 6 and performed the surgical operation with four ports, one port less than usual. There was marked tissue fibrosis in the upper mediastinal area, especially on the right side, which was thought to represent the effects of chemoradiotherapy on the tumor. Left upper mediastinal lymph node dissection was omitted to preserve vocal cord function. To determine the incision line, the esophagus was partially opened and we checked the clip placed at the location of the SCJ before chemoradiotherapy, and chose the slightly oral-side line from the clip as the oral incision line. Although the free space in both the thorax and abdomen was narrow due to scoliosis, and the convex curve of the thoracolumbar spine somewhat hindered visualization and manipulation, the surgery could be completed using the normal procedure (Fig. 6). The operative time was 6 h and 17 min, and blood loss was 70 ml.

Postoperative course: In addition to a postoperative intravenous drip, enteral nutrition was administered through the gastrostomy. One week after surgery, an otolaryngologist confirmed that swallowing function was normal. A few days after oral intake was started, the patient required fasting and administration of antimicrobials for 8 days due to increased inflammation, which was thought to be caused by aspiration due to reflux. This treatment resulted in resolution of the inflammation, and the patient’s progress was good after resumption of oral intake. He was discharged home approximately 4 weeks after surgery. The postoperative pathological diagnosis was pT3N2M0 Stage IIIB as defined by the eighth edition of the Union for International Cancer Control (UICC) tumor-node-metastasis classification scheme, and the patient is scheduled to receive postoperative chemotherapy at the previous hospital.

**Fig. 7:**
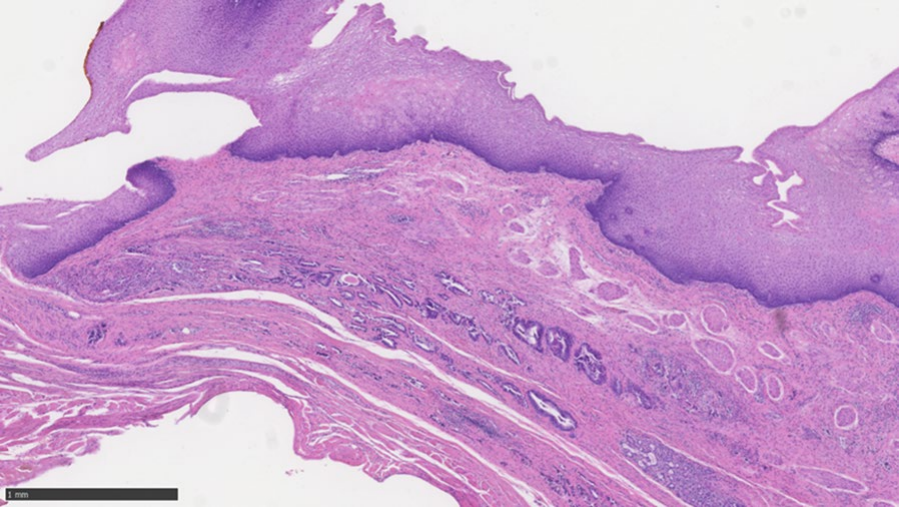
Histopathology images: tumor cells were scattered in the deep area near the oral margin. No cancer cells are seen in the squamous epithelium covering the superficial layer, suggesting that squamous re-epithelialization has occurred over the lesion

Pathologic examination: Well-differentiated adenocarcinoma-like tumor cells infiltrated the outer membrane with a depth of T3, and tumor cells were scattered in the deep area near the oral margin (Fig. 7). True oral margins were difficult to assess. The distal margin was negative. The chemotherapy response was Grade 1b as defined by the Japanese Classification of Esophageal Cancer (12th Edition). Three lymph node metastases were observed (#105, #106recR, #109L).

## Discussion

Here, we report on a patient with advanced esophageal cancer and severe scoliosis who safely underwent salvage surgery. It has been reported that larger Cobb angles are associated with more severe the restrictive ventilatory disturbance in patients with scoliosis [2]. While this patient had severe restrictive ventilatory disturbance, it did not significantly affect the perioperative course. In this case, the free space in the lower mediastinal cavities was narrow, making it difficult to secure the visual field, and forceps operation was limited by the convex curve of the thoracolumbar spine when dissecting the lower thoracic esophagus. Due to the minimal space in the thoracic cavity, there were few positions for port insertion, and surgery had to be performed with a smaller number of ports than usual. However, minimally invasive surgery was completed with generally normal procedures using thoracoscopy and laparoscopy.

Scoliosis has been reported to cause reflux esophagitis. The severity of scoliosis and the leftward scoliosis convexity are risk factors for the development of gastroesophageal reflux disease [1], and thus this patient was considered to be at high risk of reflux esophagitis. The underlying mechanisms may be that a high degree of scoliosis decreases the volume of the abdominal cavity and increases abdominal pressure, and reflux is more likely to occur as a result of deformation of the esophageal hiatus [5].

Patients with long-segment Barrett's esophagus (LSBE) are known to have a higher risk of developing cancer than those without Barrett’s esophagus or those with short-segment Barrett's esophagus (SSBE) [6]. The patient in this case had LSBE and developed esophageal cancer despite his young age. After initiation of a PPI, Barrett's esophagus regressed and the SCJ migrated anorectally. While PPIs are thought to be effective in leading to Barrett's esophagus regression and reducing the risk of cancer, there are reports that regression of Barrett's esophagus results from the reformation of squamous epithelium on top of existing columnar epithelium [7]. If the surface is replaced by squamous epithelium after cancer development and tumor reduction with chemotherapy or radiotherapy, cancer remnants may be present in the layer beneath the squamous epithelium. In the present case, tumor cells remained in the deep area where Barrett's esophagus had regressed and squamous re-epithelialization had occurred, suggesting the need for caution when determining the line of resection.

In this case, reflux symptoms caused by scoliosis were expected to persist postoperatively. Posterior mediastinal reconstruction, intrathoracic anastomosis, and total gastric tube reconstruction in patients who undergo esophagectomy have been reported to be correlated with postoperative reflux symptoms [8, 9]. Therefore, we chose to perform reconstruction with a narrow gastric tube via the anterior chest wall. Although the patient experienced postoperative bile reflux and developed aspiration pneumonia, the pneumonia resolved after the administration of antimicrobial agents, and the patient was discharged home.

## Conclusions

We report a patient with advanced esophageal cancer and severe scoliosis who safely underwent minimally invasive salvage esophagectomy. Minimally invasive esophagectomy is considered to be a viable treatment option for esophageal cancer patients with severe scoliosis.

## Data Availability

The data that support the findings of this study are available upon request from the corresponding author, Kotaro Yamashita. The data are not publicly available because they contain information that can compromise the privacy of the research participants.

## Abbreviations

PPI: Proton Pump Inhibitor
CF: Cisplatin and Fluorouracil
VC: Vital Capacity
FEV: Forced Expiratory Volume
SCJ: Squamocolumnar Junction
SUV: Standardized Uptake Value
UICC: Union for International Cancer Control
LSBE: Long-Segment Barrett's Esophagus
SSBE: Short-Segment Barrett`s Esophagus
18F-FDG: 18F-fluorodeoxyglucose
